# Breaking Barriers: Advancements in CNS Drug Delivery for Glioblastoma

**DOI:** 10.3390/medsci14010073

**Published:** 2026-02-05

**Authors:** Nicole Al Fidawi, Cecile Z. Attieh, Lara Baghdadi, Chahine El Bekai, Safaa Sayadi, Ghassan Nabbout, François Sahyoun, Hilda E. Ghadieh, Sami Azar, Frederic Harb

**Affiliations:** Faculty of Medicine and Medical Sciences, University of Balamand, Kalhat, Tripoli P.O. Box 100, Lebanon; nicole.alfidawi@std.balamand.edu.lb (N.A.F.); cecile.attieh@std.balamand.edu.lb (C.Z.A.); chahine.elbekai@std.balamand.edu.lb (C.E.B.);

**Keywords:** glioblastoma, blood–brain barrier, drug delivery systems, nanoparticles, convection-enhanced delivery, focused ultrasound, personalized medicine

## Abstract

Glioblastoma is known as the most aggressive primary brain tumor in adults, and it is still largely not curable, with a median survival of approximately 15 months when standard multimodal therapy is applied. The standard treatment nowadays is maximal safe surgical resection, associated with radiotherapy and temozolomide. Treatment effectiveness is limited not only by an impassable blood–brain barrier (BBB) to drug delivery to the brain, but also by the heterogeneity of the tumors and intrinsic or acquired drug resistance, resulting in a certain and inescapable tumor relapse. Therefore, novel drug delivery systems are being designed to overcome the BBB and improve therapeutic efficacy. These approaches include nanoparticle-mediated delivery systems, convection-enhanced intra-tumoral infusion, implantable drug-releasing devices, and noninvasive focused ultrasound technology, which induced transient disruption of the BBB. These approaches are designed to enhance local drug exposure and reduce systemic toxicity with promising preclinical and early clinical results. However, many clinical and technical challenges remain, especially the need for safety, homogeneous drug delivery, and translation of these advances into effective clinical therapies. Current glioblastoma treatment landscape and opportunities include maturing delivery systems, novel therapeutic approaches, including targeted molecular therapies and immunotherapy, as well as personalized regimens. This multidisciplinary modality may have the capacity to help not only patients with GBM but others as well through a multimodal approach of targeted drug delivery and innovative therapy in the long run to improve clinical outcomes of GBM in patients.

## 1. Introduction

**Figure 1:**
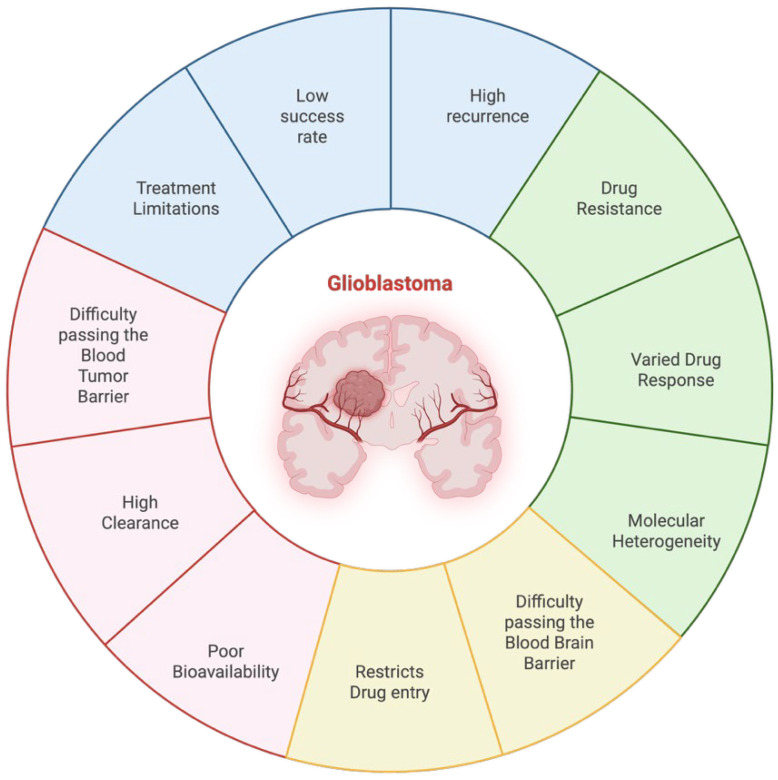
**Major obstacles contributing to the therapeutic failure of glioblastoma.** Glioblastoma remains one of the most treatment-resistant malignancies due to a combination of biological, anatomical, and pharmacological barriers. Key challenges include high recurrence rates, poor therapeutic success, intrinsic and acquired drug resistance, inter- and intra-tumoral molecular heterogeneity, and variable patient response to standard agents. In addition, systemic therapies are limited by anatomical constraints, such as the blood–brain barrier and the blood–tumor barrier, which restrict drug entry, reduce bioavailability, and increase clearance, ultimately impairing drug delivery to the tumor site.

Drug delivery to the central nervous system (CNS) is a major challenge because of its complex protective mechanisms, such as the blood–brain barrier (BBB) and blood–tumor barrier (BTB). These barriers are critical in regulating homeostasis as they have selectivity to restrict harmful substances from easily accessing brain structures. However, these protective properties also significantly limit therapeutic agent penetration and the treatment of CNS disorders, such as brain tumors [1] (Figure 1). Despite great progress in the fields of neurosciences, molecular biology, and pharmacology, the success rate for central nervous system (CNS) drug development is largely disappointing. Only ∼8.2% of drug candidates advance to clinical use, mainly resulting from problems such as poor bioavailability, high clearance of the compound in vivo, and difficulty in crossing the blood–brain barrier (BBB). Therefore, the development of drug delivery systems for CNS diseases such as GBM is important to enhance drug efficacy and patient survival. Emerging drug delivery approaches, such as nanotechnology-based systems, convection-enhanced delivery, and ultrasound-mediated BBB disruption, hold promise in addressing these challenges [2]. Through better nanoparticle design, more clinically relevant models, and sophisticated imaging and analytical procedures, recent research validates previous findings while improving them, allowing for a more accurate assessment of delivery efficiency and therapeutic relevance [3].

One of the most prevalent and malignant tumors in the central nervous system is glioblastoma (GBM). Glioblastoma growth causes a disorganized blood–tumor barrier (BTB), which is characterized by irregular vessel structure, uneven endothelial integrity, and regional variations in tight junction and transporter expression. In contrast, the healthy blood–brain barrier (BBB) is extremely impermeable and tightly controls molecular and cellular passage into the central nervous system. This causes diverse medication penetration and uneven therapeutic distribution across the tumor mass because some tumor locations have leaky, permeable vasculature, while others maintain intact BBB-like characteristics [4]. All tumors originating from the brain’s intrinsic glial cells and supporting tissue are classified as GBM. Every year, about 19 out of 0.1 million people worldwide receive a diagnosis of primary brain tumors and central nervous system (CNS) cancers. Approximately 17% of these diagnosed patients have GBM [5,6]. Although considered an uncommon disease, glioblastoma (GBM) is still a highly fatal illness. On average, patients survive only 12 to 15 months after initial diagnosis [7]. GBM is characterized by a rapid proliferation of cells and extensive migration of tumor cells into the surrounding brain, making it impossible to fully remove the tumor [8]. Due to disorganized angiogenesis, loss of tight junction integrity, and disrupted endothelial–perivascular interactions, the BTB in glioblastoma displays highly abnormal, leaky, and heterogeneous vasculature. This results in regional variability in permeability, which makes it difficult to deliver therapy to the tumor mass consistently [9]. Whereas improvements have been made in current treatment modalities, such as radiotherapy, temozolomide (TMZ) chemotherapy and maximal safe surgical resection, glioblastoma still presents a high recurrence rate. This is largely due to intra-tumoral molecular heterogeneity, restrictive function of the blood–brain barrier (BBB) and the ability of the tumor to evade host immunity by means of a localized immunosuppressive environment, all serving as major obstacles for an effective treatment [10].

Solving the issues in drug delivery to glioblastoma multiforme (GBM) is critically important for enhancing therapeutic effectiveness and survival among patients. One of the major obstacles is the blood–brain barrier (BBB), which prevents the passage of most therapeutic drugs into the brain, reducing the efficacy of standard therapy [11]. In addition, the molecular heterogeneity of GBM leads to drug resistance and tumor recurrence because cancer cells in the same tumors might have different responses to therapy [12]. Recent advancements in nanotechnology have developed smart nanocarriers which can cross the BBB and target tumor cells specifically, providing a promising way for improved drug delivery [13]. Moreover, EVs have risen to the fore as safe and natural drug delivery vehicles (DRVs) that provide direct access to GBM cells with a therapeutic load while escaping immunological clearance and biological blockade [14]. Overcoming these delivery challenges is essential to developing more effective GBM therapies and improving long-term clinical outcomes.

The evolution and selection of therapy-resistant subclones as well as the intrinsic phenotypic plasticity of tumor cells, which allow for dynamic transitions between cellular states that survive cytotoxic stress and repopulate the tumor after treatment, are increasingly recognized as the factors driving GBM recurrence [15]. Drug-resistant glioblastoma stem cells show enriched efflux transporter signatures. Up-regulation in resistant GSCs correlates with stemness and chemo-resistance. These adaptive processes are exacerbated by up-regulation of drug efflux transporters, such as members of the ATP-binding cassette family, which actively reduce intracellular drug accumulation and further limit temozolomide efficacy [16]. The poor penetration and retention of chemotherapeutic agents across the blood–brain/blood–tumor barriers are caused by a combination of resistant subpopulations, increased efflux activity, and enhanced plasticity. This undermines the efficacy of the Stupp regimen and promotes rapid relapse in treated patients [15,16].

## 2. Understanding Glioblastoma Drug Delivery Updates

### 2.1. Current Treatment Limitations in Glioblastoma Therapy

The standard of care for patients who have been newly diagnosed with glioblastoma is the Stupp protocol. This protocol consists of maximal safe resection and radiotherapy with concurrent and adjuvant TMZ (a mono-alkylating agent). Since most recurrences of glioblastomas occur within 2–3 cm from where the first lesion was, maximal safe resection is guaranteed to enhance survival rates regardless of the patient’s age and the molecular status of the tumor. There are multiple brain mapping techniques which are used before the operation to help enable safe resection. These techniques are functional MRI, navigated transcranial magnetic stimulation (nTMS), magneto-encephalography, and diffusion tract imaging. In addition, multiple tools are used during surgery to help improve the degree of resection and to diminish residual tumor volume. One of those tools is fluorescence-based visualization of the tumor using 5-aminolevulinic acid (5-ALA). However, despite the clinical benefits of 5-aminolevulinic acid (5-ALA), its use is associated with several limitations, including a heightened risk of false-positive fluorescence signals, substantial cost, potential toxicity, and challenges related to its administration. Moreover, its diagnostic utility is primarily confined to high-grade gliomas, limiting its effectiveness in detecting lower-grade tumors. Therefore, it is often combined with intraoperative ultrasound (ioUS) or intraoperative MRI (ioMRI) for optimal results. Although carmustine wafers may be applied during surgery to prolong survival, they are not part of the standard protocol due to limited efficacy, safety concerns, and tolerability issues [17].

### 2.2. Comparative Analysis of Traditional vs. Innovative Approaches

**Figure 2:**
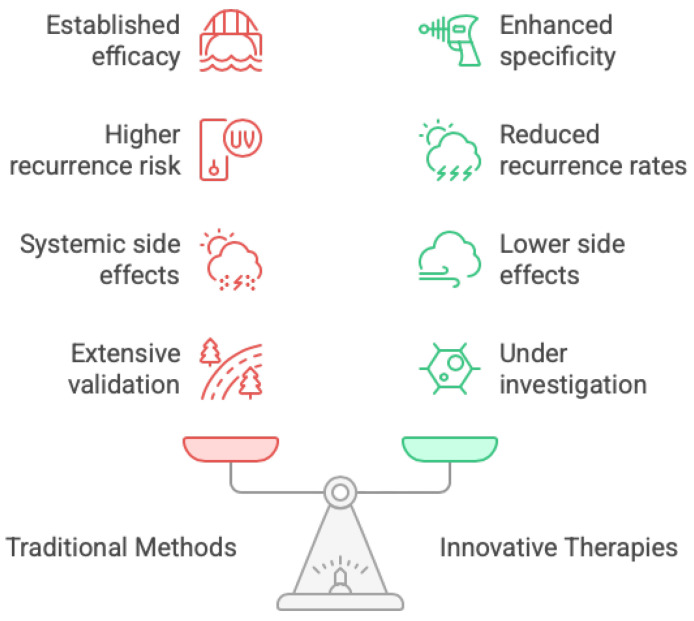
**Balancing glioblastoma treatment approaches.** Comparison of traditional methods (established efficacy, extensive validation, but higher recurrence risk and systemic side effects) versus innovative therapies (enhanced specificity, potential for lower side effects and reduced recurrence, yet still under investigation). The scale illustrates weighing proven benefit against emerging precision for patient-tailored care.

As mentioned above, the current traditional methods to treat glioblastomas are those that make up the Stupp protocol, which are surgery, radiation, and chemotherapy (Figure 2). The surgery aspect, which may be considered the most critical in GBM therapy, involves maximal safe resection of the tumor. However, it is quite challenging to resect the tumor fully due to its aggressive nature, and this is what makes the recurrence of glioblastomas strongly probable. The second aspect of the Stupp protocol is postoperative radiation therapy. Both surgical resection and radiotherapy contribute to improved overall survival in GBM patients. Additionally, radiotherapy offers the added advantage of aiding in the control of residual tumor growth following surgical intervention [17]. Finally, the last aspect of the Stupp protocol is chemotherapy, specifically TMZ administration. TMZ, in combination with radiotherapy, enhances survival rates. Unfortunately, the effectiveness of TMZ varies widely among different patients, as some patients may develop resistance to TMZ. Along with those disadvantages come the common side effects, such as fatigue, nausea, immunosuppression, and an overall decrease in the patient’s quality of life, which accompany most chemotherapeutic drugs [18].

**Figure 3:**
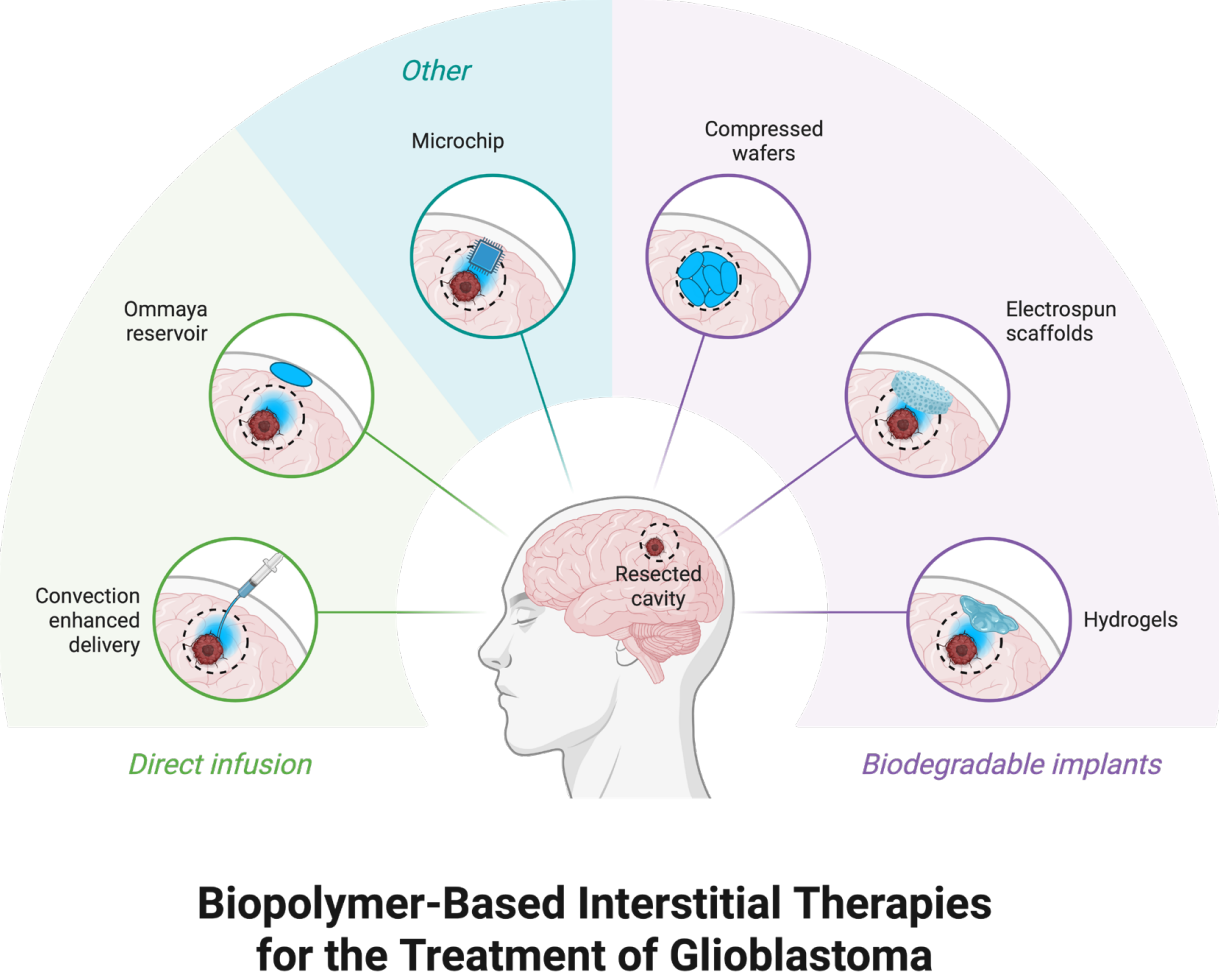
**Novel drug-delivery systems for glioblastoma.** Schematic overview of local drug-delivery strategies applied directly into the surgical resection cavity of glioblastoma. Approaches are grouped into direct infusion systems (e.g., convection-enhanced delivery catheters and Ommaya reservoirs) and biodegradable implantable systems (e.g., hydrogels, electrospun scaffolds, and compressed polymer wafers). Other emerging strategies include implantable programmable microchips allowing controlled spatiotemporal release of therapeutics. Created with Biorender (www.biorender.com).

In addition to the Stupp protocol, there have been developments in the GBM therapy, which led to new innovative treatment techniques, some of which are targeted therapy, immunotherapy, and combination therapies (*cf.* Figure 3) [18]. Targeted therapy consists of targeting specific molecular pathways involved in the development of glioblastomas. For example, drugs like Erlotinib, which are epidermal growth factor receptor inhibitors, can be used to interrupt the signaling pathways that are necessary for the tumor’s growth. Another example is the drug Bevacizumab, which is a vascular endothelial growth factor inhibitor that inhibits the angiogenesis of the tumor leading the tumor to have no blood supply and hence diminishing its growth [19]. One main issue faced with targeted therapy is the heterogeneity of glioblastomas, implying that its effects may not be generalized among the population of patients with GBM [18]. Another innovative technique is immunotherapy, which aims to enrich the body’s immune system’s ability to recognize and attack tumor cells. This can be carried out by using drugs such as Nivolumab, which is a checkpoint inhibitor. This class of drug works by blocking proteins whose normal role is to prevent the immune cells from attacking cancer cells, so inhibiting these proteins allows for more immune cells to ablate cancer cells. Immunotherapy can also be delivered by Chimeric Antigen Receptor (CAR) T Cell therapy. This method includes the ex vivo editing of T cells to express specific receptors that allow the T cells to identify and attack glioblastoma tissue. But immunotherapy has its limitations. First, the glioblastoma microenvironment is immunosuppressive and may prevent the development of productive anti-tumor immunity. Another major problem that we have encountered, as it occurs in targeted therapies as well, is the very high heterogeneity of glioblastomas [20]. This heterogeneity makes it difficult to implement treatments valid for all patients, given that therapeutic responses can strongly vary between patients with diverse molecular and genetically imbalanced tumors.

Another touchstone intervention is that of combination therapy. Combinatorial therapy aims to reach an effective combination of multiple treatment techniques, which range from traditional chemotherapy or radiotherapy to new and emerging options, such as immunotherapy or targeted therapies. This multimodal strategy is expected to act by synergism to enhance its therapeutic effects and overcome resistance encountered with mono-therapies [17]. Nonetheless, as with other treatments, combined therapy is not without drawbacks. A limitation of this construct is the potential for enhanced toxicity due to a combination of therapeutic approaches. It is therefore important for patients to be carefully monitored and managed during their course of treatment to maintain safety and tolerability [18]. A comparison of the traditional and innovative treatments suggests that, based on low specificity, conventional treatments are more likely to instigate recurrences, whereas, due to their high selectivity and specificity, novel therapies have a greater potential in specifically destroying glioblastoma cells and consequently in lowering the rate of recurrence. Moreover, novel therapies in general have a better side effect profile since they are targeted agents and carry less risk of systemic adverse effects (although it can happen). A second, more fundamental distinction lies in the depth of clinical validation. Conventional therapeutic modalities are underpinned by extensive, rigorously controlled trials that delineate both efficacy and safety across large, heterogeneous patient cohorts, whereas extracellular-vesicle-based interventions currently lack comparable evidence. In contrast, innovative treatments are still under investigation and may produce variable responses depending on individual patient characteristics.

## 3. Overview of Recent Breakthroughs in Drug Delivery Methods for Glioblastoma

### 3.1. Nanoparticles and Nanotechnology

Treating, diagnosing, and managing gliomas has undergone a revolution due to the field of nanotechnology. This change is mostly ascribed to more recent developments in bioengineering, easier access to medications, and the capacity to specifically target cancer cells. Because of their small size, large surface area, unique structural features, binding affinity, ability to penetrate cell membranes or tissues, and long elimination half-life in the circulation, nanoparticles (NPs) are becoming more and more used in the field of cancer therapeutics and diagnostics. Their high surface-to-volume ratio enhances their therapeutic effectiveness by delivering tiny biomolecules like proteins, nucleic acids, and medications to the target region. Drug transport across the blood–brain barrier is improved by a number of strategies, many of which involve disrupting the BBB. Consequently, the integrity of the cerebral microvasculature is compromised by this disruption. The administration of anticancer drugs via polymers or lipid NPs is one method that shows promise [21]. It is important to note that most of these approaches have been demonstrated primarily in pre-clinical models, and translation to the clinical setting remains limited.

Nanocarriers can traverse the BBB by utilizing endogenous transport channels like receptor-mediated and adsorptive transcytosis. Nanoparticles functionalized with ligands (such as transferrin, insulin, or low-density lipoprotein receptor targeting moieties) engage endothelial transporters and undergo vesicular transcytosis into the brain parenchyma in receptor-mediated transcytosis. The negatively charged endothelium glycocalyx and cationic nanoparticle surfaces interact electrostatically during adsorptive transcytosis. Because of their extended circulation, enhanced specificity, and controlled release capacities, these modified transport pathways are being studied more and more for glioma-targeted administration. They can improve targeted BBB penetration while maintaining barrier integrity [22]. Nanoparticle-based delivery systems offer several general benefits that make them appealing for glioma therapy, in addition to facilitating BBB passage. While ligand functionalization enables selective targeting of tumor cells or BBB receptors, hence boosting drug accumulation at the disease site, their nanoscale size and surface engineering enable longer circulation durations by minimizing quick renal clearance and opsonization. Additionally, regulated or stimuli-responsive drug release can be achieved with nanoparticles, improving intracellular drug availability and reducing off-target toxicity. When compared to traditional systemic chemotherapy, these characteristics together result in increased therapeutic efficacy and a better safety profile [23].

The use of certain nanoparticles in glioblastoma monotherapy has given promising results.

Eugenio et al. demonstrated that silver/silver chloride nanoparticles (Ag/AgCI-NPs) significantly inhibited the proliferation of GBM02 glioblastoma cells, with efficacy surpassing that of temozolomide, particularly at higher nanoparticle concentrations. Kesinostat, a histone deacetxase inhibitor, can cause apoptosis, improve cell differentiation, and induce cell cycle arrest, but has limited application due to inadequate delivery. Nevertheless, Kesinostat encapsulation in poly (D, L-lactide)-b-methoxy poly (ethylene glycol) nanoparticles produced favorable results, increasing the survival rates of experimental animals [21]. Human serum albumin nanoparticles containing chlece-aluniou phthalocyanine (AlCIPc) on U87MG cells were used to investigate various light sources in photodynamic treatment. Apoptosis was the major mode of cell death in all cases, according to the flow cytometry analysis [24].

Reaching the tumor site without harming the healthy tissues is one of the major problems in cancer treatment. The BBB is the primary obstacle to medication delivery in malignant gliomas.

Additionally, P-glycoprotein lowers the drug’s effective concentration by pumping the utilized medications out of the cell using ATP. It is possible to combine medications used to treat glioblastoma into NPs and functionalize them with different ligands to allow them to cross and target the BBB. Apart from appropriate administration, medication stability rises, and unfavorable side effects are somewhat decreased [21]. Moreover, Table 1 compares the IC_50_ values of different nanoparticle formulations across various glioblastoma cell models.

As summarized in Table 1, Ag/AgCl nanoparticles consistently show lower IC_50_ values than free temozolomide across glioblastoma models, while demonstrating reduced toxicity toward primary astrocytes. This enhanced cytotoxic efficacy is likely due to improved cellular internalization, time-dependent intracellular accumulation, and sustained antiproliferative effects of nanoparticles, which may partially stop resistance mechanisms associated with conventional temozolomide therapy.

Nanocarriers can be engineered from a variety of materials, including organic compounds, metals, minerals, and polymers. These nanosystems have been utilized to deliver a range of anticancer agents, such as temozolomide, paclitaxel, docetaxel, cisplatin, doxorubicin, curcumin, and nucleic acids directly to the brain, enhancing drug bioavailability and targeting efficiency.

Despite their therapeutic promise, however, there remain serious safety concerns. Importantly, a critical translational barrier remains the unresolved risk that certain nanoparticle formulations may act as carcinogenic initiators or induce genotoxic injury. Further studies are needed to address these concerns to safely translate nanoparticle-based therapeutics into the clinic [21]. In vitro studies have demonstrated that exposure to certain nanomaterials can induce genetic aberrations, including DNA damage and chromosomal abnormalities, particularly with metal and metal oxide nanoparticles, quantum dots, and fibrous nanomaterials. Such genotoxic effects may disrupt normal cell cycle regulation, promote genomic instability, and ultimately contribute to malignant transformation. Given the ability of small nanoparticles to enter cells via endocytosis and interact directly or indirectly with genetic material, further systematic evaluation of their long-term carcinogenic potential and safety is essential before widespread clinical application [25].

### 3.2. Convection-Enhanced Delivery (CED)

Passage of the blood–brain barrier (BBB) is one of the common problems in drug delivery to gliomas. An approach that bypasses the BBB and allows for direct distribution within the brain is convection-enhanced delivery (CED), which can deliver drugs to the brain that are normally blocked by this barrier [26]. The CED process is designed to confine more than 99% of the therapeutic payload within the tumor bed, effectively sparing the surrounding brain from exposure by circumventing the blood–brain barrier rather than traversing it. To accomplish this, a stereotactic infusion cannula is accurately aimed at the target, and a controlled positive pressure difference is used to distribute the infusate across the brain parenchyma. In contrast, most traditional drug delivery systems largely depend on a passive diffusion mechanism where molecular size directly dictates tissue penetration and distribution—and can thereby impose significant obstacles for larger therapeutic agents. On the other hand, CED offers a significant advantage in that drug distribution, which is predominantly influenced by hydrostatic pressure gradients as opposed to molecular size, allows for the delivery of a broader range of therapeutic agents [27].

The device, which usually delivers therapy in a substantially spherical manner, dispenses at approximately 0.5 to 10 µL/min. Unlike single injection application, which targets up to five millimeters from the tip of the catheter, CED allows distribution of therapy up to 6 cm from the tip of the catheter and provides an over-4000-fold increase in volume of distribution. In addition, its directed application and fast delivery, CED minimizes the toxicities of systemic drug injection by offering highly specific tumor bed targeting [28].

Numerous clinical trials were conducted to evaluate the effectiveness of CED because of its advantages. Following the identification of the interleukin-13 receptor (IL-13R) as a therapeutic target and the creation of an IL-13-based cytotoxic drug in the early 2000s, one of the major CED trials took place. This receptor is a desirable target for selective therapy since it is significantly elevated in malignant glioma cells but only slightly expressed in normal brain tissue. A modified pseudomonas exotoxin and IL-13 are combined in a recombinant fusion molecule known as IL-13–PE38QQR (cintredekin besudotox). Animal models indicated tumor necrosis without substantial systemic toxicity, while preclinical research showed preferential binding and cytotoxicity toward GBM cells with little damage to normal brain tissue. The most effective use of the medication was when it was administered directly into the tumor using convection-enhanced delivery (CED). Early phase I/II clinical investigations in patients with newly diagnosed or recurring malignant gliomas were supported by these findings. These trials established acceptable safety profiles and revealed moderate survival gains, which eventually led to the phase III PRECISE experiment. In adults with their first glioblastoma recurrence, the phase III PRECISE trial assessed convection-enhanced administration of IL-13–PE38QQR against Gliadel wafers, an active standard comparator. This was the first phase III randomized controlled trial in glioblastoma to compare a recognized conventional treatment with a therapeutically active drug administered via CED. The findings showed therapeutic equivalency but not superiority, since the overall survival in the CED arm was similar to that attained with the FDA-approved Gliadel Wafers therapy [26].

The lack of tumor IL-13 receptor expression-based patient selection and the technical difficulties associated with CED, such as significant catheter positioning variability, were the study’s limitations. Nearly half of treated patients had suboptimal catheter placement, which probably hindered effective drug delivery despite central review, stereotactic guidance, and standardized training. Adverse events were similar between groups and consisted mainly of localized neurological impairments, cerebral edema, altered mental status, and thromboembolic consequences. Collectively, these findings underline that although CED can theoretically increase intra-tumoral drug distribution, clinical effects in human trials have been variable, mostly due to backflow, variability in catheter placement, and inadequate or unequal drug dispersion within the tumor bed [26].

Despite the fact that CED has shown substantial superiority in drug delivery, several factors should be considered during its application. The procedure associated with CED catheter insertion may contribute to intra-cerebral edema. Accordingly, the majority of clinical protocols limit tumor size to 4 cm and exclude patients with posterior fossa tumors because larger lesions are often associated with neurological deterioration. In these cases, the excess volume from infusions can deteriorate intracranial pressure as undesirable rather than being beneficial. Catheters are placed 1–2 cm from the subarachnoid spaces and resection cavities and no closer than 0.5 cm to the ependymal surface in an effort to ease complications and improve the delivery accuracy. Furthermore, the catheter material needs to be tailored such that it has appropriate stiffness to maintain trajectory yet flexibility required for accurate delivery to a desired target location [26].

Proceeding to the technical enhancements required for improving CED (cf. Table 2), a significant issue remains, which is the backflow of infused material along the cannula track, especially at high flow rates. This restriction happens when the pressure differential between the catheter tip and tumor tissue reaches an equilibrium, and a retrograde mode occurs with diluted drug against targeted drug delivery [26]. A solution to this problem has been to provide a stepped cannula. This altered cannula has a stepped design that acts as an anatomical back-stop, minimizing the risk of retrograde flow in both the body of the cannula and along the outer luminal tract [27]. These developments are necessary to improve both dose accuracy and therapeutic potential.

Human CED methods have incorporated real-time imaging techniques to track infusion as it happens. The feasibility of evaluating contrast distribution intraoperatively and modifying cannula placement or infusion parameters to enhance tumor coverage has been shown in phase I studies of CED with liposomal irinotecan co-infused with gadolinium contrast and viewed under MRI. A significant drawback of static planning is addressed by such MR-guided CED, which enables immediate assessment of infusate spread, early detection of backflow or maldistribution, and dynamic adjustment during the procedure. When taken as a whole, computational simulations and real-time MRI monitoring represent significant advancements toward more reliable and efficient CED, mitigating poor distribution caused by backflow, catheter placement variability, and unpredictable tissue transport that have led to inconsistent outcomes in earlier human studies [29].

### 3.3. Implantable Drug Delivery Devices

Implantable drug delivery devices (IDDDs) are a newly developed strategy to improve the treatment of GBM by enabling localized pharmacotherapy and compensating for the blood–brain barrier (BBB) hindrance. A well-known example is the Gliadel^®^ wafer, a biodegradable polymer containing carmustine (BCNU) that is implanted directly into the resection cavity after surgery for sustained local chemotherapy. It has been shown to increase survival compared with systemic chemotherapy only in clinical trials [30].

Rigid polymeric implants, such as Gliadel^®^ wafers, which contain carmustine embedded in a biodegradable polyanhydride matrix and inserted into the surgical resection cavity, have historically dominated implantable local delivery solutions for glioblastoma. Although this method proved that cerebral chemotherapy is clinically feasible, these rigid implants present several inherent drawbacks. Their mechanical stiffness creates a significant mismatch with soft brain tissue, leading to uneven contact with surrounding parenchyma, poor conformability to irregular resection cavities, and suboptimal drug dispersion by cerebrospinal fluid diffusion. Additionally, release kinetics are characterized by an early burst despite slower bulk degradation of the polymer, and drug penetration is mostly diffusion-limited, usually extending only a few millimeters beyond the implant surface. The invasiveness of such implantable devices also introduces biocompatibility and infection risks that mandate rigorous surgical procedures and follow-up handling. Collectively, these limitations hinder the ability to adjust to tumor heterogeneity, restrict effective medication coverage, and undermine overall therapeutic efficacy [8,9,10,11,12,13,14,15,16,17,18,19,20,21,22,23,24,25,26,27,28,29,30,31,32].

The progress in microfabrication technology has made it possible to implant multifunctional microdevices with capabilities of delivering multiple therapeutic agents concomitantly, which offer a full-spectrum approach for glioblastoma (GBM) therapy. For instance, 3D-printed patient-specific implants mediating the controllable release of DNA-nanocomplexes have been applied to the localized GBM treatment, indicating an opportunity for personalized medicine in this field [31].

Novel approaches are now attempting to avoid these issues by developing soft, biodegradable implants that can conform to the brain’s complex form and allow for more accurate drug delivery with less safety risk for the patient. For example, hydrogel-mediated systems have been engineered to invigorate chimeric antigen receptor (CAR) T cells in situ within the tumor microenvironment, which is a novel strategic innovation for treating GBM [33].

Implants made of soft, biodegradable hydrogel have become a more biomimetic option. To minimize mechanical mismatch and enhance tissue integration, hydrogels can be designed to closely resemble the mechanical characteristics of brain tissue and to conform to intricate hollow geometries. Long-term sustained and adjustable release patterns are made possible by their highly hydrated networks, which provide more control over diffusion and degradation kinetics. Crucially, hydrogels provide significantly greater payload versatility, allowing for the use of viral vectors, biologics, nanoparticles, or small-molecule chemotherapeutics. Additionally, they can be made for combinatorial delivery, which enables the sequential or simultaneous release of several medicines from a single implant. Building on these ideas, many of the pharmacokinetic and physical limitations that restrict inflexible wafer-based systems are addressed by microfabricated and composite implanted devices, which further increase control over spatiotemporal drug delivery [8].

Despite their therapeutic potential, implantable drug-delivery devices have significant surgical and regulatory restrictions because they must be implanted intracranially during surgery and are linked to risks of wound-healing complications, infection, and local inflammation. Seizures, intracranial hypertension, meningitis, cerebral edema, poor neurosurgical wound healing, and water migration are some of the major local side effects of gliadel when compared to a placebo. These factors have prevented widespread clinical adoption [8].

Although IDDDs are a promising battleground against GBM, further investigation is needed to improve formulations, biocompatibility, and clinical usability.

### 3.4. Focused Ultrasound

Focused ultrasound (FUS) is a method using concentrated beams of ultrasonic energy for highly precise targeting in the depths of the brain while avoiding damage to surrounding normal tissue. Once in the specific area, these FUS waves can cause several effects on tissues. First, high-intensity acoustic energy is sent to the tumor using focused ultrasound (FUS) to induce a sudden increase in temperature in the local region. This elevation in temperature results in irreversible cellular injury with either coagulative necrosis of tumor cells (i.e., tumor ablation). Thus, FUS achieves a tumor volume reduction with direct destruction of cancerous tissue and preservation of neighboring non-cancerous structures.

Furthermore, FUS can also improve the delivery of drugs to the tumor (Table 3). It achieves this by briefly breaking down the blood–brain barrier (BBB) to let drugs into the tumor, where they can act on cancer cells. FUS is a safe approach to avoid complications related to invasive methods. It also allows the treatment to be seen while it is happening and makes it possible to adjust the procedure, if needed. Moreover, because FUS employs spatially confined acoustic energy, selectively engaging the target tissue while sparing adjacent neural and perilesional structures, the possibility of damaging healthy brain tissues around stimulated areas is reduced, which means decreasing the probability of side effects.

Although a promising modality for the treatment of various diseases, focused ultrasound (FUS) faces a few obstacles, and anatomic barriers are one of them. Because the skull is a dense and inhomogeneous material, it can block efficient ultrasound propagation, which could cause distortion, attenuation, or energy losses before reaching the focused position in brain tissue. This limitation may prevent the accuracy and effectiveness of FUS, especially in treating deep-seated or centrally located brain tumors. Moreover, breach of the blood–brain barrier may induce inflammation, causing adverse effects and making recovery more complex [34].

However, the effectiveness of focused ultrasound is influenced by patient-specific skull anatomy because the thickness, density, and heterogeneity of the skull can attenuate or distort ultrasonic transmission, thorough pre-treatment calibration is necessary to guarantee proper energy deposition and prevent overheating or off-target consequences. Furthermore, patients differ in the degree and consistency of blood–brain barrier opening, which may affect the effectiveness of drug administration and raise the possibility of inflammation or other negative effects.

Early phase I MR-guided FUS studies in recurrent glioblastoma have included a limited number of participants in the cohort. These studies mainly showed safety and feasibility, but there was no clear increase in progression-free or overall survival when compared to previous expectations.

### 3.5. Electrochemotherapy

Electrochemotherapy (ECT) is a treatment that combines electroporation (EP), which involves the use of electrical pulses, with the injection of a chemotherapeutic drug. The electrical pulses open pores in the cell membrane, allowing increased influx and efflux of molecules. Normally, cancer cells typically exhibit poor drug uptake, so using this technique increases the uptake of anticancer drugs into the cancer cells [35].

Electroporation can be broken down into three stages at the level of a single cell: First, the induction step, where the field-induced membrane potential difference reaches the critical threshold value. Second, the expansion step, where membrane defects continue to exist if the field is present. Third, the resealing step, where the membrane repairs, which is necessary to maintain cell viability [36]. Patients can receive medication in ECT through two major methods. The medicine can be administered intravenously or intra-tumorally, which involves injecting the drug directly into the tumor [35]. Administration of drugs during both the pulse and slow resealing phases results in a major increase in drug intracellular concentration. This amount is multiplied almost 100-fold when compared to conventional chemotherapy [36].

Regarding the brain, ECT is mainly applied to the blood–brain barrier to make it more permeable to drugs that are otherwise unable to cross it. However, intra-tumoral ECT is still under study. Bleomycin and Cisplatin are charged drugs that have shown high therapeutic efficacy in tumors when used with ECT, but they are impermeable to the blood–brain barrier. So, making the BBB more permeable using this technique would create opportunities for effective drug therapies [37]. Regarding glioma treatments, application of intra-tumoral ECT in rat glioma showed a 69% complete response rate, where 9 out of 13 rats had tumor regression. However, MRI data exposed necrosis to the area subjected to the treatment, as well as some fluid-filled cavities. It was demonstrated that some risks associated with the use of ECT include edema, hemorrhage, infection, or epileptic events prompted by excitation of surrounding tissue [37].

The use of ECT indeed showed to have many benefits, but it is also important to note that it poses many challenges and risks, such as bleeding, seizures, and tissue damage, which add to the importance of precise pulse parameters and to the fact that the use of ECT for brain tumors is still experimental and is pending regulatory approval [38].

### 3.6. Immunotherapy and Targeted Therapies

Recently, Immunotherapy and targeted therapies have emerged, aiming to specifically disrupt molecular abnormalities within GBM cells, potentially enhancing therapeutic effectiveness while reducing side effects [39]. One promising approach is anti-angiogenic therapy, particularly using bevacizumab, a monoclonal antibody against vascular endothelial growth factor (VEGF). Although bevacizumab extends progression-free survival in patients with recurrent GBM, it has not significantly improved overall survival in newly diagnosed cases, suggesting limitations due to compensatory angiogenic mechanisms [40,41]. This limited efficacy is largely due to adaptive tumor responses, including activation of alternative pro-angiogenic pathways, enhanced hypoxia-driven invasiveness, and vascular co-option that bypasses VEGF dependence. As a result, glioblastoma proliferation persists despite VEGF inhibition, highlighting the part that compensatory angiogenic processes and tumor plasticity play in treatment resistance [40].

The targeting of the epidermal growth factor receptor (EGFR), which is often amplified or mutated in GBM, is one such important therapeutic approach. Although preclinical data strongly suggested that erlotinib, gefitinib, or cetuximab would be effective against HNSCC 2-4, clinical trials have only shown modest effects. This partial success may be due to molecular heterogeneity of GBM and the existence of compensatory signaling pathways [30,42]. This reduced efficacy can be attributed to significant intra-tumoral molecular variability, such as varying EGFR amplification, extracellular-domain mutations, and loss of EGFR expression in resistant clones, by reducing consistent drug targeting. Additionally, tumor cells can avoid EGFR reliance by activating compensatory signaling pathways, such as PI3K/AKT and MET, which reduces clinical responses to EGFR-directed treatments [42].

Integrins, primarily αvβ3 and αvβ5, are critical for GBM invasion and angiogenesis. The integrin inhibitor cilengitide, initially considered effective, failed to improve survival in Phase III clinical trials. This exhibits the difficulty translating preclinical successes to clinical efficacy due to glioblastoma angiogenic and invasive pathway redundancy, which decreased reliance on integrin signaling, the complex and context-dependent function of integrins, which preclinical models oversimplified, and the preclinical systems’ limitations that prevented them from accurately reflecting the heterogeneity and microenvironmental adaptability of human tumors. Lastly, any advantage limited to integrin-dependent cancers was diluted due to the lack of biomarker-based patient selection, underscoring the challenge of converting preclinical success into clinical usefulness [41,43].

The tumor microenvironment (TME) is a critical driver of glioblastoma (GBM) progression and therapy resistance. Lately, efforts have been focused on modulating such microenvironments as a therapeutic approach to increase the efficacy of treatment and have focused mainly on tumor-associated macrophages and stromal elements [41,44]. Immunotherapy approaches targeting immune checkpoints (PD-1, PD-L1, CTLA-4) have also been investigated but have generally been disappointing due to GBM’s profoundly immunosuppressive microenvironment [45,46]. Current research emphasizes combination therapies, including targeted agents with immunotherapies or conventional treatments, to circumvent resistance mechanisms [42,46]. Despite significant challenges such as the blood–brain barrier, molecular heterogeneity, and resistance, targeted therapies continue to be vigorously investigated. Innovative drug-delivery methods, personalized therapeutic strategies, and combination approaches represent future directions to potentially improve patient outcomes in GBM [41,46].

## 4. Prospects and Challenges

### 4.1. Potential Future Developments in CNS Drug Delivery Research

Drug delivery across the blood–brain barrier has proven to be one of the most prominent challenges in the treatment of brain tumors. Many trends in research have been surfacing concerning this issue.

Liposomes, solid lipid nanoparticles, non-polymeric micelles, lipoplex, dendrimers, polymeric nanoparticles, polymeric micelles, nanotubes, silica nanoparticles, quantum dots, gold nanoparticles, and magnetic nanoparticles are examples of the various compositions of nanoparticles, which are solid colloidal particles of matter with sizes ranging from 1 to 100 nanometers [47]. Nanotechnology is an essential tool when developing new systems for the efficient delivery of potential therapeutic and diagnostic compounds to specific areas of the brain, because they reduce the adverse side effects associated with non-specific drug distribution, increased drug concentration at the desired site of action, and, consequently, improved therapeutic effectiveness [48]. Loading medications onto nanoparticles allows them to pass the blood–brain barrier without blocking its chemical composition. Nanoparticles’ physicochemical and biomimetic features determine their ability to traverse the BBB. Evaluating the chemical composition of nanoparticles is crucial for minimizing their toxicity when used in clinical settings. So far, evidence supports the notion of nanoparticle-assisted medication delivery [47].

Another advancement is the use of exosomes in crossing the blood–brain barrier. Extracellular vesicles (EVs) are membrane-bound nanoscale bodies secreted by almost all types of prokaryotic and eukaryotic cells. EVs are further classified as ectosomes and exosomes. Ectosomes bud directly from the plasma membrane into the extracellular matrix while exosomes form intraluminal bodies through double plasma membrane invagination from multivesicular bodies (MVBs) [49]. Exosomes can deliver both hydrophilic and hydrophobic medications, and their drug-carrying capability is exceptional. They are an excellent drug delivery vehicle for a variety of diseases, including brain disorders, due to their advantages in tumor homing ability, extended blood circulation half-life, excellent BBB traversal, lower toxicity, hypo-immunogenicity, and reflection of the “inheritance” from the parent cell and cellular affinity [49]. In recent years, artificially created exosomes have emerged as superior to natural exosomes in terms of large-scale production, uniform isolation, drug encapsulation, stability, and quality assurance. Manufactured exosomes are regarded as potentially excellent carriers for chemical and biological therapies, as we can control the circulation time and selectivity [50]. However, exosomes are not always beneficial. In fact, they may contribute to cancer metastasis to the brain. Cancer cells create many exosomes, which help cancer cells migrate, invade, and metastasize via angiogenesis. Tumor-cell-derived exosomes can interact with vascular tissues, including the brain vasculature, making them more susceptible to angiogenic stimuli and metastasis. Furthermore, exosomes generated from cancer cells carry immunosuppressive proteins that inhibit immune system processes and encourage tumor growth [50].

### 4.2. Anticipated Challenges and Hurdles in Translating Research into Clinical Applications

A study analyzing CNS drugs in clinical trials between 1990 and 2012 revealed that these drugs were 45% less likely to succeed in Phase III trials compared to non-CNS drugs. Additionally, 46% of CNS drugs failed because they did not show greater efficacy than a placebo [51]. The primary objective of the Phase III “REGAL” trial, which aimed to demonstrate an extension in progression-free survival by comparing cediranib alone, cediranib combined with lomustine, and placebo in patients with recurrent glioblastoma (GBM), was not achieved [52]. Rindopepimut, a drug targeting the EGFRvIII mutation, effectively increased patients’ progression-free survival to 10–15 months and overall survival to 22–26 months, compared to just 6 and 15 months, respectively, reported in previous studies [53]. However, it did not increase overall survival in a bigger Phase III trial, since the control group performed better than the treatment group (20.0 months vs. 20.1 months) [54]. Trials will continue despite this setback, highlighting the challenges involved in introducing novel treatments. A recent study showed that differences between the tumor microenvironment in in vitro and in vivo models create significant obstacles in developing new drugs for glioblastoma (GBM) using lab-grown models [55]. Several critical limitations must be considered when evaluating a drug’s efficacy. The drug must be delivered to the tumor effectively, achieving therapeutic levels without getting away in normal tissue. For example, the clinical trial that evaluated lapatinib was unsuccessful due to an inadequate drug level in patients [56]. Moreover, to develop drugs for CNS-alleviating diseases, it is crucial to cross the BBB (or blood–brain barrier) effectively with a balance of molecular size and lipid-solubility. Intra-tumoral injections may solve this problem, but it has critical shortcomings in that they are time-consuming, not widely accepted by oncologists and raise biosafety concerns.

A retrospective analysis of EGFR inhibitors offers some understanding of why the latter did not pan out in clinical GBM trials. These agents were effective in the laboratory setting, but EGFR inhibitors, including erlotinib and gefitinib, have not led to better clinical responses. This shortfall has been attributed to inadequate CNS accumulation. Efflux pumps, such as P-glycoprotein and ABCG2, actively expel the compounds from the brain parenchyma, preventing attainment of therapeutic levels [57,58].

In addition to pharmacokinetic hurdles, recent progress in the development of drugs for GBM has encountered formidable barriers. Low clinical trial accrual (in part by classifying GBM as an orphan disease) has limited the statistical confirmation of modest, yet clinically important improvements. Further difficulties involve ensuring an appropriate definition of clinical endpoints, obtaining efficient patient stratification for prognostic factors and choosing a relevant control arm [56].

Furthermore, the risk and cost of surgery make monitoring molecular features in brain tumors more difficult. It is therefore of utmost importance to establish non-imaging methods to evaluate drug efficacy, also because some therapies could decrease tumor growth and not its overall size [18].

To minimize the limitations stemming from the challenge of being able to have tumor cells and accurately mimicking GBM biology, multiple systems were developed, such as the use of patient-derived glioma stem cells and human embryonic stem cell (derived cerebral organoids), allowing for a better replication of tumor heterogeneity [59]. In addition, the use of in-silico mathematical modeling showed allows for better prediction and understanding of drug delivery and tumor development [60].

Stratifying patients by their different molecular subtypes and using biomarkers, whose absence led to multiple failed trials, would allow for better trial designs and improve the therapeutic success rates. Also, combining different drug delivery methods, integrating immunotherapy with BBB opening, and enhancing carrier design to exploit tumor vasculature are all methods to help face the multiple challenges and hurdles faced in translating the research into clinical applications.

Finally, allowing for the collaboration between engineers, clinicians, and pharmacologists would allow for an enrichment in the advancements that are being carried out and would allow for a better understanding of the model, yielding better therapeutic results.

## 5. Conclusions

The different modalities that were stated throughout this paper are all signs of advancements in the current and future management of GBM and provide complementary approaches to overcome the BBB/BTB. Drug-loaded NPs were found to be able to selectively deliver the drug into AD hallmark amyloid plaques. In the same way, nanoparticles stimulate drug penetration into brain tissue through the EPR (enhanced permeability and retention) effect and represent a major step forward in the treatment of glioblastoma. The efficacy of nanoparticles depends mainly on their physical characteristics and size, which can determine the capability of nanoparticles to penetrate biological barriers and exert the intended therapeutic effect [3].

Contrastingly, despite encouraging preclinical results, there are limited clinical investigations of the potential for nanoparticle integration in radiation oncologic practice. The enormity of the challenge here comes because there are many different types of nanoparticles that have been considered in the research community, and they can differ drastically, including mass (size), geometry (shape) and surface functionality. This diversity adds a level of complexity in interpreting their uptake mechanisms, the biological pathways for radiation sensitization, extracorporeal clearance and potential toxicologic attributes. Therefore, substantial in-depth preclinical research is required to discover the best nanoparticle formulations and dosing schedules suited for this clinical development. Despite encouraging preclinical data, several unresolved issues continue to obstruct clinical translation and limit real-world utility [61].

To date, the major roadblocks have been keeping track of vital molecular pathways and finding novel drugs, which are targeted. The introduction of molecular profiling and patient stratification techniques into routine clinical practice offers hope that future trials with rationally targeted therapies may demonstrate improved results. The development of novel treatments largely relies on appropriate identification of therapeutic targets, suitable drug candidates and dependable biomarkers for patient selection in early clinical studies [62].

In conclusion, the use of personalized regimens, combination strategies, and rigorous clinical translation provides us with a clear understanding of the drug delivery model and helps improve the outcome of patients with GBM.

## Figures and Tables

**Table 1:** Summary of IC_50_ values and proliferation effects of Ag/AgCl nanoparticles and temozolomide in glioblastoma in vitro.

Treatment	Model System	IC_50_ (µg/mL) at 24 h	IC_50_ (µg/mL) at 48 h	IC_50_ (µg/mL) at 72 h	Population Doubling Time (PDT) at Selected Doses	Comments
**Ag/AgCl-NPs**	GBM02 glioblastoma cells	6.3 ± 0.3	4.6 ± 0.3	2.3 ± 0.3	At 2.5 µg/mL, PDT increased from 20 h (control) to 107 ± 34 h; at 5.0 µg/mL, PDT increased to 2514 ± 1673 h	Rapidly decreases IC_50_ over time and strongly prolongs cell doubling; suggests accumulation of nanoparticles in tumor cells.
	Astrocytes (primary human)	18.5 ± 0.8	8.4 ± 0.6	9.1 ± 0.3	At 5.0 µg/mL, PDT was 340 ± 240 h	Higher IC_50_ and slower proliferation inhibition indicate lower toxicity to normal brain cells.
**Temozolomide (TMZ)**	GBM02 cells	198.1 ± 9.4	18.3 ± 1.1	8.8 ± 0.5	PDT increased to 499 ± 106 h at 80 µg/mL	TMZ required much higher concentrations to achieve similar IC_50_ and still had substantial toxicity to astrocytes.
	Astrocytes	49.5 ± 2.9	45.2 ± 2.4	60.3 ± 3.4	At 80 µg/mL, PDT was 1120 ± 210 h	Normal astrocytes are more sensitive to TMZ than to Ag/AgCl-NPs; emphasizes the therapeutic window of nanoparticles.
**Ag/AgCl-NPs from *Kaempferia rotunda & Zingiber mauritiana***	Glioblastoma stem cells (GSCs)	–	–	IC_50_ = 6.8 µg/mL (*K. rotunda*) and 10.4 µg/mL (*Z. mauritiana*)	–	At 32 µg/mL, these biogenic nanoparticles inhibited 71–77% of GSC growth; complete inhibition was achieved at 40 µg/mL, illustrating their potency against stem-cell populations.

**Table 2:** Advances in convection-enhanced delivery (CED).

Innovation	Description of Catheters/Devices	Quantitative Improvement	Comments and Citation
**Hollow-fiber catheters with micro-openings**	Catheters incorporate millions of tiny (0.45 µm) openings along the shaft. Their design allows distributed infusion rather than a single outflow port	Increased the amount of infusate delivered to the target brain tissue by up to threefold compared with conventional end-port catheters and reduced backflow	Improves uniform distribution of drugs during CED; still experimental and requires careful control of infusion rates.
**Multi-port catheters (Cleveland Multiport Catheter)**	A central cannula with several flexible micro-catheters extending into brain tissue; each micro-catheter has its own infusion port	Showed minimal backflow in porcine models but had lower infusion rates compared with single-needle catheters	May permit simultaneous delivery of multiple agents or coverage of irregularly shaped tumors, but technical complexity remains.
**Real-time imaging and distributed infusion**	Use of MR imaging or stereotactic guidance during CED to track infusate distribution	Enables adjustment of infusion parameters to maximize volume of distribution and avoid leakage	Quantitative improvements vary by study but generally report larger volumes of distribution and more homogeneous infusion.

**Table 3:** Summary of focused ultrasound-mediated enhancement of chemotherapeutic delivery in glioblastoma models.

FUS Intervention	Model System/Experiment	Drug and Dosing	Quantitative Effect	Notes and Citation
**FUS + etoposide (pre-clinical)**	Orthotopic mouse model of glioblastoma	Etoposide administered systemically; FUS applied to sonicate the tumor	FUS increased etoposide tumor-to-serum ratio by 3.5-fold and intra-tumoral concentration by >8-fold; tumor growth at day 14 was reduced by 45% vs controls; median survival improved from 19 to 25 days	A single FUS treatment transiently disrupted the BBB; adding repeated treatments or combining with other drugs may further enhance efficacy.
**FUS-enhanced paclitaxel delivery (clinical/pre-clinical)**	Patients and rodent models with GBM or brain metastases	Paclitaxel with low-intensity pulsed ultrasound	FUS increased paclitaxel concentrations in tumor tissue by 3.7-fold relative to baseline	Studies used MR-guided FUS; limited sample sizes.
**FUS-enhanced carboplatin delivery**	Rodent models	Carboplatin ± FUS	Tumor carboplatin concentration increased 5.9-fold after FUS-mediated BBB opening	Demonstrates that hydrophilic drugs can also benefit from FUS.
**FUS-enhanced temozolomide delivery**	Pre-clinical (rodent)	Temozolomide at therapeutic doses	Performing BBB opening concomitantly with temozolomide administration increased its brain concentration by 7.7-fold	Suggests that timing of ultrasound relative to drug administration is critical; may enable dose reductions while maintaining efficacy.

## Data Availability

No new data were created or analyzed in this study.
